# Combining blue native polyacrylamide gel electrophoresis with liquid chromatography tandem mass spectrometry as an effective strategy for analyzing potential membrane protein complexes of *Mycobacterium bovis *bacillus Calmette-Guérin

**DOI:** 10.1186/1471-2164-12-40

**Published:** 2011-01-18

**Authors:** Jianhua Zheng, Candong Wei, Lina Zhao, Liguo Liu, Wenchuan Leng, Weijun Li, Qi Jin

**Affiliations:** 1 State Key Laboratory for Molecular Virology and Genetic Engineering, Institute of Pathogen Biology, Chinese Academy of Medical Sciences & Peking Union Medical College, Beijing, PR China

## Abstract

**Background:**

Tuberculosis is an infectious bacterial disease in humans caused primarily by *Mycobacterium tuberculosis*, and infects one-third of the world's total population. *Mycobacterium bovis *bacillus Calmette-Guérin (BCG) vaccine has been widely used to prevent tuberculosis worldwide since 1921. Membrane proteins play important roles in various cellular processes, and the protein-protein interactions involved in these processes may provide further information about molecular organization and cellular pathways. However, membrane proteins are notoriously under-represented by traditional two-dimensional polyacrylamide gel electrophoresis (2-D PAGE) and little is known about mycobacterial membrane and membrane-associated protein complexes. Here we investigated *M. bovis *BCG by an alternative proteomic strategy coupling blue native PAGE to liquid chromatography tandem mass spectrometry (LC-MS/MS) to characterize potential protein-protein interactions in membrane fractions.

**Results:**

Using this approach, we analyzed native molecular composition of protein complexes in BCG membrane fractions. As a result, 40 proteins (including 12 integral membrane proteins), which were organized in 9 different gel bands, were unambiguous identified. The proteins identified have been experimentally confirmed using 2-D SDS PAGE. We identified MmpL8 and four neighboring proteins that were involved in lipid transport complexes, and all subunits of ATP synthase complex in their monomeric states. Two phenolpthiocerol synthases and three arabinosyltransferases belonging to individual operons were obtained in different gel bands. Furthermore, two giant multifunctional enzymes, Pks7 and Pks8, and four mycobacterial Hsp family members were determined. Additionally, seven ribosomal proteins involved in polyribosome complex and two subunits of the succinate dehydrogenase complex were also found. Notablely, some proteins with high hydrophobicity or multiple transmembrane helixes were identified well in our work.

**Conclusions:**

In this study, we utilized LC-MS/MS in combination with blue native PAGE to characterize modular components of multiprotein complexes in BCG membrane fractions. The results demonstrated that the proteomic strategy was a reliable and reproducible tool for analysis of BCG multiprotein complexes. The identification in our study may provide some evidence for further study of BCG protein interaction.

## Background

Tuberculosis (TB) is an infectious bacterial disease, caused primarily by *Mycobacterium tuberculosis *[1]. One third of the world's population is currently infected with TB bacillus. In many countries, immunization of infants with bacillus Calmette-Guérin vaccine (BCG) protects against TB meningitis and other severe forms of TB in children less than five years of age http://www.who.int/en/. However, BCG vaccination is not recommended for adults because the protection provided is variable [2]. The threat to human health presented by TB worldwide is increased by the emergence of multidrug-resistant strains and co-infection with human immunodeficiency virus [3]. Therefore, it is crucial to develop novel strategies to improve the BCG vaccine or to develop effective drugs to reduce the socioeconomic and health burden associated with TB.

Membrane proteins play important roles in various cellular processes, including cell adhesion, cell metabolism, ion transport and signal transduction [4]. Membrane proteins are represented by around 30% of the genome and constitute approximately 70% of all pharmaceutical drug targets [5]. Traditional one-dimensional electrophoresis (1-DE) and two-dimensional electrophoresis (2-DE) coupled with MS are commonly used to study membrane proteome [4]. However, solubility and low abundance issues of membrane proteins remain to be the main challenges in gel electrophoresis [6]. Large-scale identification of *M. tuberculosis *membrane proteins came forth since 2002 [7-10]. In 2007, Mattow *et al. *carried out membrane subproteomic analysis on *M. bovis *BCG Copenhagen by 1-D SDS polyacrylamide gel electrophoresis (PAGE) and 2-DE and obtained 125 unique proteins including 54 proteins harboring 1-14 predicted TMHs [11]. In 2008, Målen *et al. *identified 351 proteins by a combination of both gel-based and gel-free protein and peptide fractionation methods, including 103 integral membrane proteins with at least one predicted transmembrane region [12]. Recently, they also separated hydrophobic membrane and membrane-associated proteins directly from sonicated *M. tuberculosis *H37Rv using SDS PAGE and liquid chromatography tandem mass spectrometry (LC-MS/MS) [13].

In general, proteins rarely function completely independently, which makes identification of protein-protein interactions crucial to our understanding of numerous cellular processes [14]. A range of proteomic approaches have been adopted to analyze diverse protein interactions [15]. Blue native (BN) PAGE, a specialized type of native electrophoresis, is widely applied to investigate protein-protein interactions, especially for membrane proteins [16]. BN PAGE was first described in 1991 for the separation of membrane protein complexes from the respiratory chain of human mitochondria [17]. Previous studies have demonstrated that multiprotein membrane complexes can be resolved into individual blue bands in 1-D by BN PAGE, and the protein subunits of each complex can then be separated by various types of gel electrophoresis in 2-D, for example, SDS PAGE, doubled SDS PAGE (dSDS PAGE) or IEF/SDS 3-D PAGE [18]. However, identification of the stained protein spots in 2-D PAGE by MS analysis is tedious and time-consuming, despite recent advances in automation [19]. Furthermore, artifactual methionine oxidation that occurrs during SDS PAGE cannot be entirely prevented, which is a problem when attempting to identify physiological oxidative modifications of complexes [20]. In 2005, Babusiak *et al. *presented a novel native 2-D separation technique that combined native electrophoresis with native LC and could be applied to the separation and identification of intact heme-binding protein complexes in murine erythroleukemia cells [21]. The feasibility of this approach was also illustrated by Fandino *et al. *who combined BN PAGE and non-gel shotgun methods [19]. Wessels *et al. *extended this method to analyze full length of 1-D BN gel lanes and identified potentially interacting proteins by protein correlation profiling (PCP) [14]. Since these methods are fully compatible with MS/MS protein identification, they could be widely applied in the study of membrane protein complexes.

Studies on a variety of membrane protein complexes have been reported, however, little is known about mycobacterial membrane complexes. Here we investigated *M. bovis *var BCG NCTC 5692, an attenuated derivative of *M. bovis*, by an approach coupling BN PAGE to LC-MS/MS to analyze potential protein-protein interactions in membrane fractions. All proteins identified in 1-D BN bands were confirmed using 2-D gel. We have obtained a profile of the *M. bovis *BCG potential membrane and membrane-associated protein complexes, and these results may provide some insights into BCG protein interaction studies.

## Results and discussion

### Sample pre-treatments for BN PAGE

**Figure 1:**
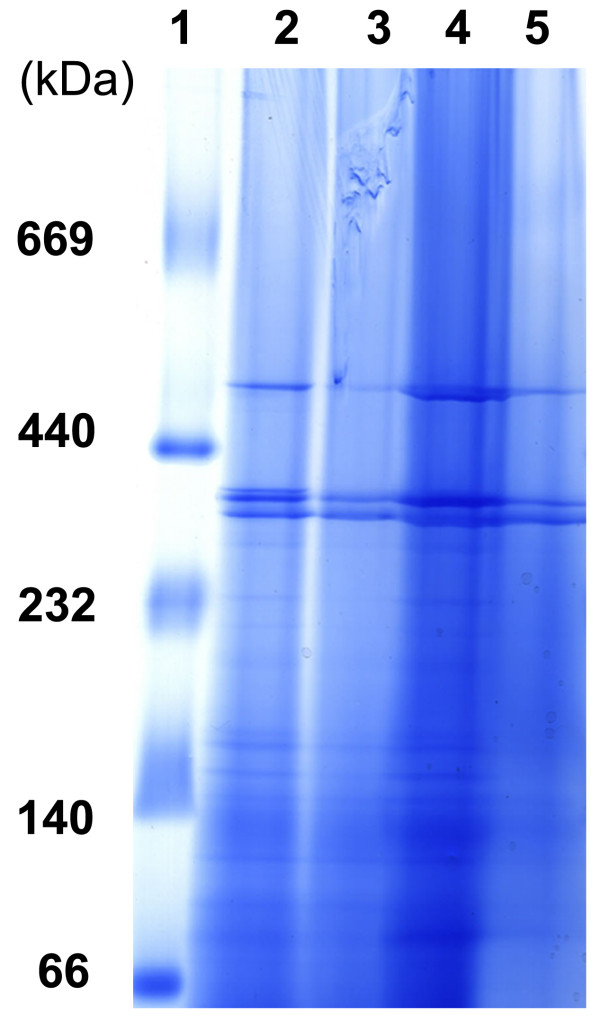
**BN PAGE of membrane fractions from *M. bovis *BCG**. Membrane fractions were incubated with detergents for at least one hour at 4°C. Lane 1 was High Molecular Weight (HMW) calibration and lane 2, 3 were treated with Triton X-100 (0.2% and 0.1% (w/v), respectively). Lane 4, 5 were solubilized with DDM (2% and 1% (w/v), respectively). Gel gradient was 4-16%. The gel was run according to the protocol given in Methods.

The aim of this work is to isolate and characterize native and structurally intact membrane protein complexes directly from BCG membrane fractions. The protein complex stability during the sample preparation and separation is an extremely important factor in effective identification. It has been demonstrated that some detergents allow for effective solubilization of proteins in oligomeric state, maintenance of enzyme activity and sharp resolution in electrophoresis [22]. Mild non-ionic detergents, Triton X-100 and n-dodecyl-β-D-maltoside (DDM), were chosen to solubilize membrane fractions and the effect of different concentrations of detergents was tested experimentally. Briefly, two samples of the extracted membrane fractions (see Methods for details) were treated with 2% (w/v) Triton X-100 to a final concentration of 0.2% and 0.1%, respectively, while another two samples were resolved with DDM to a final concentration of 2% and 1% (w/v), respectively. The migration of each sample treated with different detergents and concentrations was compared and the best result was obtained with 0.2% (w/v) of Triton X-100 (Figure 1). This concentration appeared to represent a good compromise, avoiding both the partial disintegration of native complexes caused by excess detergent, and the appearance of gel band smearing due to insufficient concentrations of detergent.

### Separation by BN PAGE and SDS PAGE

**Figure 2:**
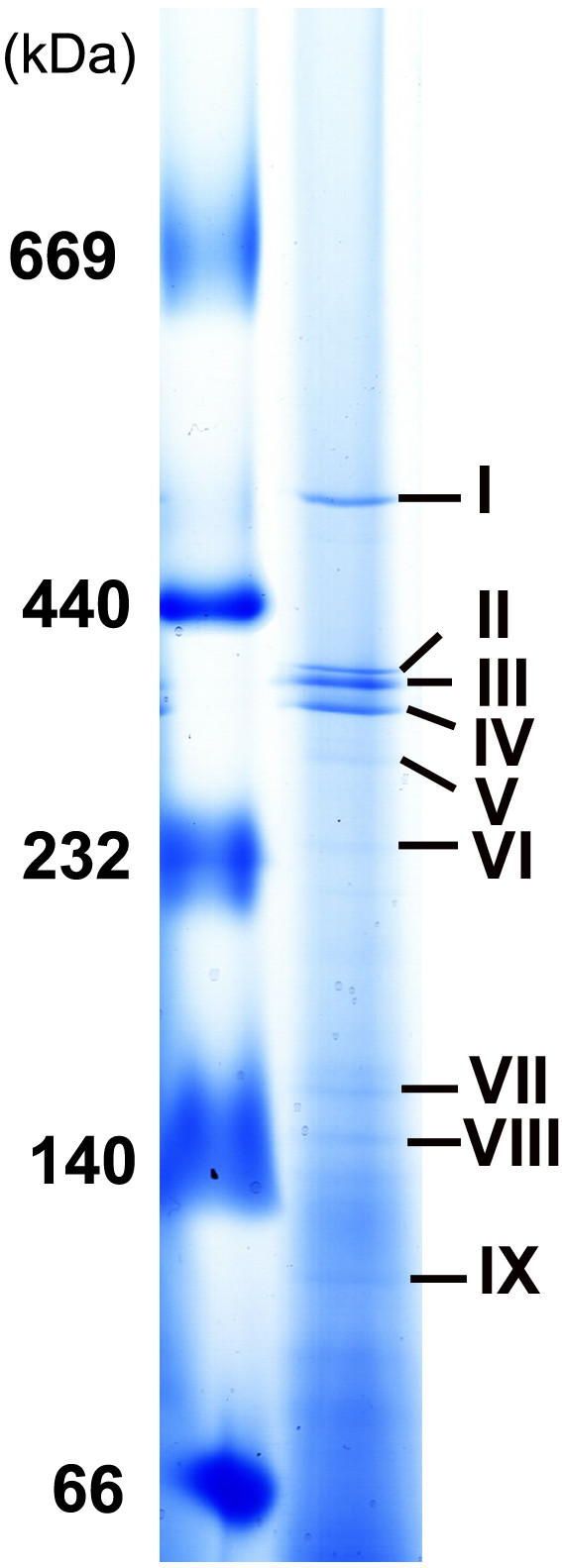
**BN PAGE separation of *M. bovis *BCG membrane fractions treated with 0.2% Triton X-100**. Gel gradient was 4-16% and the gel was stained with CBB G-250. Left lane was High Molecular Weight (HMW) calibration, and right was migration of BCG membrane fractions.

In the present study, biological membranes were solubilized with 0.2% (w/v) Triton X-100 and separation by BN PAGE. According to their size, proteins migrated more slowly with increased running distance and decreased pore size of the gradient gel and stopped almost entirely when they approached their size-dependent specific pore-size limit [16]. Figure 2 shows a representative BN PAGE map of membrane fractions. To determine the protein composition in BN gel bands, nine bands with high staining intensity were excised and the peptide mixtures derived from them were individually subjected to LC-MS/MS system as described in the experimental procedures. Finally, proteins were identified by database search and replicate measurements have confirmed the identity of these protein hits.

The result showed that each BN band represented a protein complex. Totally, nine potential multiprotein complexes were obtained. The blue band I and band VII contained some subunits of lipid metabolism complex, whereas the sharp band II was identified as ATP synthase. In band III and IV, two members of phenolpthiocerol synthase and three of Emb protein complex were found, respectively. In band V two polyketide synthases were recognized, whereas band VI contained four members of heat shock protein family. In addition, several members of polyribosomal proteins were found in band VIII. In the last band IX, two subunits of the succinate dehydrogenase (SDH) enzyme complex were identified.

In 2008, Målen *et al. *obtained a comprehensive picture of the *M.bovis *BCG membrane protein repertoire [12]. Of these proteins identified in their study, four protein complexes were found, including ATP synthase, NAD(P) transhydrogenase, ubiquinone oxidoreductase and ubiquinol-cytochrome *C *reductase. In detail, the soluble protein encoded by the *pntAa *gene has been shown to be associated with two other proteins encoded by *pntAb *and *pntB *genes, forming NAD(P) transhydrogenases complex on the membrane. Additionally, six subunits of the ATP synthase complex, five subunits of the ubiquinone oxidoreductase complex and three subunits of the cytochrome *bc1 *complex were also identified, respectively.

**Figure 3:**
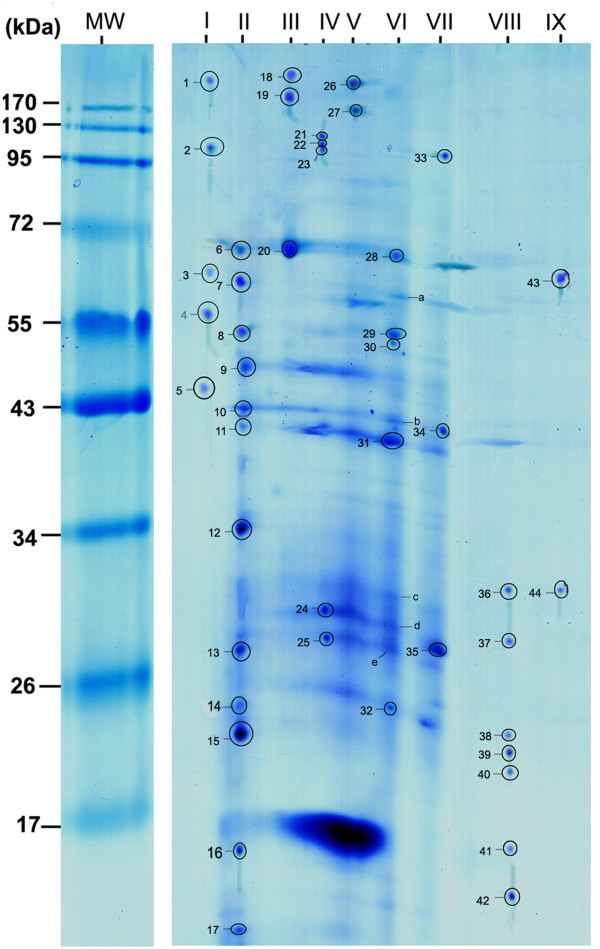
**2-D SDS PAGE map of multiprotein complexes from *M. bovis *BCG membrane fractions**. Nine BN bands were loaded on top of a 12% separating gel and separated by SDS PAGE. 2-D gel was stained with colloidal Coomassie blue and left lane was PageRuler Prestained Protein Ladder (Fermentas). Roman numerals on top of the map indicated BN gel bands. The spots were excised and identified by MS/MS and the proteins were shown in Table 1.

In the present study, to confirm the protein composition of each complex, nine bands which cut out from 1-D BN gel were resolved into their individual subunits by denaturing SDS PAGE. The subunits of each complex were ordered according to their molecular weight in vertical rows. Figure 3 showed a 2-D PAGE map of protein complexes identified in BN gel bands and 44 spots were recognizable on the map. In total, 40 distinct proteins were identified by two or more peptide hits using the Mascot algorithms. A complete list of all identified proteins is provided in Table 1 and Additional file 1. On average, the identified proteins matched with more than six unique peptides. Of these proteins, 12 proteins had at least one transmembrane helix (TMH) predicted by TMHMM 2.0 (excluding the possible signal sequences predicted using SignalP). The average GRAVY value, which is used to evaluate the hydrophilicity and hydrophobicity of a protein along with its amino acid sequence, was 0.33 for integral membrane proteins (IMPs) [23]. Based on the Pasteur Institute functional classification tree, the identified proteins were distributed across several functional groups (see Table 1 for details). Most of the identified proteins were involved in intermediary metabolism and respiration (Functional category 7, 27.5%). Additionally, seven proteins (17.5%) were required for lipid metabolism (Functional category 1), information pathways (Functional category 2) and cell wall and cell processes (Functional category 3), respectively. It was interesting that some proteins with high hydrophobicity involved in complexes were identified in our study, for example, EmbC, EmbA and EmbB, which contained 12 TMHs. Therefore, our method may offer an effective alternative strategy to identify proteins with high hydrophobicity or multiple TMHs in complex. To investigate the potential protein complexes, the molecular composition and function of the proteins were analyzed as discussed below.

**Table 1:** List of *M. bovis *BCG potential multiprotein complexes and proteins identified in this study.

**protein^a**)**^**	description	peptide^b)^	SC^c)^	p*I*^d)^	M*r*(kDa)^e)^	score^f)^	TMH^g)^	Gravy^h)^	function^i)^	band^j)^	spot^k)^
Pks2	polyketide synthase	10	4.09	5.18	227.13	60.02	0	-0.001	1	I	1
MmpL8	integral membrane transport protein	3	4.78	9.85	116.28	35.23	12	0.330	3	I	2
FadD23	fatty-acid-CoA ligase	4	8.56	5.42	62.84	29.57	2	0.064	1	I	3
PapA1	polyketide synthase associated protein	4	11.35	5.33	56.13	27.47	0	0.013	1	I	4
BCG_3890c	putative transposase	2	3.19	11.94	46.11	31.02	0	-0.577	5	I	5
LpqW	putative lipoprotein	3	4.25	5.23	66.37	104.53	0	0.027	3	II	6
AtpA	ATP synthase alpha chain	14	22.60	4.89	59.48	1883.30	0	-0.207	7	II	7
AtpD	ATP synthase beta chain	19	46.50	4.71	53.18	2917.93	0	-0.168	7	II	8
AtpH	ATP synthase delta chain	9	18.40	5.28	48.83	285.95	1	0.027	7	II	9
LipO	putative esterase	4	36.07	10.59	46.10	36.07	0	-0.113	7	II	10
AtpG	ATP synthase gamma chain	9	21.30	5.26	33.93	593.00	0	-0.267	7	II	12
AtpB	ATP synthase a chain	2	12.80	6.14	27.51	80.91	5	0.818	7	II,IV	13,25
AtpF	ATP synthase b chain	6	39.80	5.11	18.32	115.66	1	0.040	7	II	15
AtpC	ATP synthase epsilon chain	5	47.10	4.55	13.13	43.53	0	-0.061	7	II	16
AtpE	ATP synthase c chain	2	49.40	4.78	8.06	24.67	2	1.016	7	II	17
PpsC	phenolpthiocerol synthesis type-I polyketide synthase	8	5.30	4.92	231.22	59.56	0	0.028	1	III	18
PpsA	phenolpthiocerol synthesis type-I polyketide synthase	12	7.25	5.02	199.69	132.56	0	-0.045	1	III	19
EmbB	integral membrane indolylacetylinositol arabinosyltransferase	4	2.55	10.14	118.14	40.51	12	0.312	3	IV	21
EmbC	integral membrane indolylacetylinositol arabinosyltransferase	7	6.31	10.42	117.83	59.83	12	0.246	3	IV	22
EmbA	integral membrane indolylacetylinositol arabinosyltransferase	3	3.56	10.12	115.92	39.95	12	0.338	3	IV	23
BCG_2759c	putative membrane alanine rich protein	4	12.22	12.05	29.06	98.48	2	0.113	10	IV	24
Pks7	polyketide synthase	12	8.09	5.29	221.75	154.53	0	0.213	1	V	26
Pks8	polyketide synthase	6	4.50	5.21	167.47	41.55	0	0.264	1	V	27
DnaK	chaperone protein	11	26.72	4.70	66.79	202.48	0	-0.368	0	VI	28
GroEL2	60 KDA chaperonin 2	5	11.30	4.70	56.69	82.72	0	-0.091	0	VI	29
GroEL1	60 kDa chaperonin 1	6	9.46	4.84	55.84	61.98	0	0.110	0	VI	30
DnaJ2	chaperone protein	3	10.21	6.24	41.03	31.23	0	-0.237	0	VI,II	11,31
FurA	ferric uptake regulation protein	2	15.33	5.46	16.53	23.78	0	0.013	9	VI	32
MmpL11	transmembrane transport protein	3	2.48	10.14	104.06	34.28	12	0.337	3	VII	33
BCG_0241c	transmembrane protein	3	6.32	10.29	45.89	20.18	8	0.323	3	VII	34
BCG_0238c	hypothetical protein	2	8.98	7.75	18.52	44.45	0	-0.292	10	VII	35
RplB	50S ribosomal protein L2	9	29.30	11.94	30.62	100.45	0	-0.851	2	VIII	36
RpsD	30S ribosomal protein S4	2	9.50	10.33	23.46	95.47	0	-0.722	2	VIII	37
RpsE	30S ribosomal protein S5	3	13.20	10.74	22.93	24.67	0	-0.171	2	VIII	38
RplE	50S ribosomal protein L5	5	19.30	10.52	21.01	73.79	0	-0.344	2	VIII	39
RplV	50S ribosomal protein L22	8	44.20	12.24	20.37	162.57	0	-0.494	2	VIII	40
RplM	50S ribosomal protein L13	2	17.00	10.41	16.34	22.52	0	-0.418	2	VIII	41
RplR	50S ribosomal protein L18	6	46.70	12.10	13.18	133.98	0	-0.329	2	VIII	42
SdhA	succinate dehydrogenase (flavoprotein subunit)	20	36.78	5.65	65.24	1062.30	0	-0.246	7	IX	43
SdhB	succinate dehydrogenase (iron-sulphur protein subunit)	4	12.55	8.93	30.01	211.54	0	-0.154	7	IX	44

a) Protein name in *M. bovis *BCG database.

b) Number of identified unique peptides obtained for a particular protein.

c) Sequence coverage in percent of the identified protein.

d) Isoelectric point of the protein that calculated from its amino acid sequence.

e) Molecular weight in kDa of the respective protein.

f) The Mascot score of the protein identification.

g) Transmembrane helices predicted by TMHMM 2.0 program.

h) Grand average of hydrophobicity predicted by ProtParam.

i) Functional classification based on the Pasteur Institute functional classification tree.

j) Number of BN gel bands for complex identification.

k) Number of spots for protein identification.

### Analysis of potential membrane and membrane-associated protein complexes

#### Lipid metabolism complex

**Figure 4:**
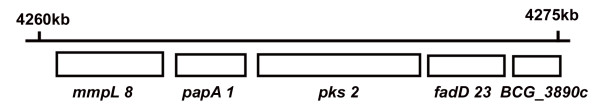
**The co-localization of *mmpL8 *gene**. The *mmpL8 *gene was linked to genes involved in polyketide biosynthesis (*pks2 *gene) and lipid metabolism (*papA1 *and *fadD23 *genes).

In BN gel, one band migrated sharply in the upper part of the gradient gel. In this band, we identified MmpL8 and the polyketide synthase Pks2, along with the proteins PapA1, FadD23 and BCG_3890c. In 2-D gel this BN gel band was resolved into five proteins (spot 1-5). Spot number 1 migrated with an apparent molecular mass of about 230 kDa and was identified as polyketide synthase. Spot 2 was identified as integral membrane transport protein (MmpL 8). In addition, a fatty-acid-CoA ligase (FadD23, spot 3), a polyketide synthase associated protein (PapA1, spot 4) and a putative transposase (BCG_3890c, spot 5) were also found in 2-D gel. Genome analysis reveals that the *mmpL8 *gene is positioned 8 kbp downstream from the *pks2 *gene, and the *papA1 *gene is also linked to the *pks2 *gene (see Figure 4 for details). The co-localization of the *mmpL *gene with genes involved in polyketide biosynthesis (*pks *gene) and lipid metabolism (*papA *and *fadD *genes), suggests a similar role of these proteins in complex lipid transport in mycobacteria [24]. Sequence analysis reveals MmpL8 protein comprises 1089 amino acid residues and is predicted to contain 12 membrane-spanning alpha helices. The PapA1 protein matched with four unique peptides and was a suspected membrane-associated acyltransferase, suggesting its involvement in the esterification of trehalose with methyl-branched fatty acids during the biosynthesis of polyacyltrehalose and SL-1 [25]. Additionally, FadD23 can combine with the protein encoded by *pks *gene in various ways to form complex hybrid metabolites [26].

This is also the case with MmpL11, which is closely related to BCG_0241c and BCG_0238c (identified in band VII). In 2-D gel, three proteins were unambiguous identified (spot 33, 34 and 35, respectively). These encoding genes occur within the same apparent gene cluster, which is conserved in all of the mycobacterial genomes sequenced to date and found to play a possible fundamental role in mycobacterial pathogenicity [27]. Since proteins MmpL8 and MmpL11 may be involved in the transportation of molecules that function in host-pathogen interactions, inhibitors of MmpL8 or MmpL11 may provide novel drug targets in the future.

#### ATP synthase complex

Gel band II comprised 11 proteins, as measured by LC MS/MS, including four IMPs. All members of ATP synthase complex were identified from this band. Resolution of the band on the 2-D gel revealed eight subunits of ATP synthase were obtained. In detail, subunit a (AtpB, spot 13), which contained five transmembrane helices, matched with two unique peptides. Subunit b (AtpF, spot 15), which embedded in the membrane and contained a long helical domain extending into the cytoplasm, was in contact with subunit a, and subunit c (AtpE, spot 17) with an apparent molecular mass of about 8 kDa, which contained two hairpin-like TMHs, was connected by a central sequence that was exposed to the cytoplasm [28]. Subunit β (AtpD, spot 8), which constituted part of the α3β3 hexamer, contained 19 peptides and the sequence coverage was 46.5%. Additionally, subunits α (AtpA, spot 7) and γ (AtpG, spot 12) consisted of 14 and nine peptides, respectively (see Table 1 for details). Subunits δ (AtpH, spot 9) and ε (AtpC, spot 16) contained nine and five unique peptides, respectively. In our work, although the stoichiometry of the α_3_β_3_γ complex could not be determined by MS/MS measurements, the numbers of the identified peptides represented, at least partially, the relative abundance of these subunits (14, 19 and nine unique peptides, respectively). In fact, using the procedure proposed by Abresch *et al.*, we were able to estimate the molecular ratios of the subunits [29].

It is a common feature of BN PAGE that minor fractions of multiprotein complexes precipitate during the electrophoretic run. In fact, spot 6 on 2-D gel (Figure 3) was identified as putative lipoprotein LpqW. Analysis of lipoprotein LpqW with bioinformatic softwares predicted a GRAVY score of 0.027 and no transmembrane helices. Interestingly, STRING 8.3 database analysis predicted that the LpqW protein is a functional partner of integral membrane arabinosyltransferase A (EmbA, identified in band IV) (confidence score > 0.6). It seemed that the LpqW protein was a cytoplasmic protein that interacted with the Emb protein complex (identified in band IV). Additionally, the putative esterase (LipO, spot 10) migrated as "free" proteins because of no attachment to other protein subunits using STRING database analysis. Spot 11 was identified as chaperone protein (DnaJ2), which was a subunit of heat shock protein family (identified in band VI). Spot 14, aligned with other proteins in band II, was observed but no identification was obtained by MS analysis.

#### Phenolpthiocerol synthase complex

In band III on BN gel, we obtained two members of phenolpthiocerol synthase (PPS) protein family, which was encoded by a large operon [30]. In 2-D gel spot 18 and 19 were identified as PpsC and PpsA, and they matched with eight and 12 unique peptides, respectively. A previous study demonstrated that PpsC was involved in a late step in the biosynthesis of phthiocerol and phenolphthiocerol and disruption of PpsC in *M. bovis *BCG led to be an inability to produce mycoside B and phthiocerol dimycocerosates (PDIMs) [31]. Practically, since the proteins encoded by *pps *genes are unique to pathogenic mycobacteria, they may be used as targets in the high throughput screening of desperately needed antimycobacterial drugs. Additionally, database search after MS/MS analysis of pigmented spot 20 did not yield any result.

#### Emb protein complex

BN gel band IV was subjected to in-gel digestion and analyzed by LC-MS/MS. Three proteins encoded by *emb *genes, EmbA, EmbB and EmbC, were identified in this band. In 2-D gel, three spots (spot 21, 22 and 23) were identified as arabinosyltransferases (EmbB, EmbC and EmbA, respectively). On the basis of sequence analysis, these proteins are IMPs with 12 TMHs (excluding the possible signal peptide), and match with four, seven and three unique peptides, respectively. Functional analysis showed that EmbC is involved in the synthesis of lipoarabinomannan, and EmbA and EmbB are responsible for the polymerization of arabinose into the arabinan of AG [32]. They have been proposed to be the main targets of ethambutol (EMB). Disruption of these proteins would inhibit the formation of the mycolyl-arabinogalactan-peptidoglycan complex and may lead to increased permeability of the cell wall and drug uptake [33]. Therefore, they may serve as targets for the development of therapeutic approaches against TB.

In this band, a putative membrane alanine rich protein was obtained (BCG_2759c, spot 24). Analysis of the primary sequence of the protein predicted two transmembrane helices and STRING database analysis predicted that the protein BCG_2759c was no interaction among those proteins identified in our study. In this way, we were able to conclude that the protein BCG_2759c was a cytoplasmic membrane protein and a "free" protein because of no clear attachment to other protein identified in 2-D gel. In addition, spot 25 was identified as subunit a of ATP synthase complex (identified in band II).

#### Polyketide synthases

Two polyketide synthases (PKSs), Pks7 and Pks8, were identified in BN band V. Genome analysis reveals that *pks7 *and *pks8 *gene are located in a cluster of *pks *genes. The proteins encoded by these genes are large multifunctional enzymes and belong to the same PKS family required for PDIM synthesis in mycobacteria [34]. In 2-D gel, spot 26 migrated with an apparent molecular mass of great than 200 kDa was identified as Pks7, and the protein matched with 12 unique peptides. It has been reported that Pks7 plays an important role in virulence during the persistent phase of infection [35]. Additionally, Pks8 (spot 27) matched with six peptides, and has been implicated in the biosynthesis of monomethyl-branched unsaturated fatty acids [36].

#### Heat shock protein family

Heat shock proteins (Hsps) are involved in a highly conserved family, and first recognized by their upregulated expression in response to host exposure to raised temperatures [37]. In the present study, we identified GroEL1, GroEL2, DnaK, DnaJ and FurA in gel band VI (see Table 1 for details). In 2-D gel, spots 29 and 30 were identified as the 60 kDa chaperonin 2 (GroEL 2) and 1 (GroEL 1), respectively. It has been reported that 60 kDa chaperonins are one type of molecular chaperones found on the surface of cells and these proteins have high affinity for lipid monolayers and bilayers and can associate with lipid membranes [38]. Accordingly, it was no great surprise to detect the presence of two 60 kDa chaperonins (GroEL1 and GroEL2) in the membrane fractions.

Additionally, DnaJ (spot 31) is a stress gene-encoding protein that is highly conserved among bacterial genera. Therefore, gene *dnaJ *is useful not only for identification of mycobacterial species but also for inferring their phylogenetic relationships [39]. Suzanna *et al. *found that during the folding of some proteins, DnaK (spot 28) recognized extending polypeptide chains and cooperated with DnaJ in stabilizing this intermediate conformation [40]. In addition, spot 32 was identified as a ferric uptake regulation protein (FurA). On the basis of bioinformatics analysis, FurA was a cytoplasmic protein and no interaction with Hsp family members. Thus, the protein migrated as "free" proteins in 2-D gel.

#### Polyribosomal proteins

In general, ribosomes are ribonucleoprotein particles consisting of two subunits, which are designated as the small subunit (30S) and the large subunit (50S) in bacteria [41]. Ribosomal proteins involved in the subunits are the major components of the polyribosome complex where mRNA is translated into protein [9]. In this study, seven ribosomal proteins were detected in band VIII, including five proteins in the large ribosomal subunit (L2, L5, L13, L18 and L22, see Table 1 for details) and two in the small ribosomal subunit (S4 and S5). All these proteins identified in BN gel were confirmed using 2-D SDS gel. Despite the lack of predicted TMHs, some ribosomal proteins are associated with the plasma membrane by IMPs and may interact transiently with the nascent membrane or secreted proteins [33]. It is important to note that some ribosomal proteins can easily dissociate from polyribosomal complex during their preparation and separation.

#### Succinate dehydrogenase complex

Members of succinate dehydrogenase (SDH) complex were found in band IX. SDH is a membrane-bound protein complex and key enzyme participating in intermediary metabolism and aerobic energy production in bacteria [42]. This complex is composed of four subunits SdhA/B/C/D. Here we were only able to identify two subunits: SdhA and SdhB (spot 43 and 44, respectively). MS analysis indicated that SdhA matched with 20 peptides, which was the highest number of unique peptide reported in our study. Intriguingly, these two subunits can exhibit SDH activity in the absence of SdhC/D, which are the membrane components [43].

In addition to the proteins discussed above, some spots were observed in 2-D gel but no identifications were found by MS analysis. For example, there were five spots presented in band VI (spot a, b, c, d and e, see Figure 3 for spots without circles) which showed exogenous stains on 2-D denaturing gel, while no proteins were determined among these spots. The spots without identifications also presented in other bands (including band IV, V and VII, spots were not showed in Figure 3). This observation may suggest that some pigment molecules can bind to gel without destaining completely.

## Conclusions

In the present study, by coupling LC-MS/MS to BN PAGE we were able to unambiguously identify potential membrane/membrane-associated protein complexes in BCG membrane fractions. The proteins identified in complexes were confirmed using 2-D gel. For example, eight subunits of ATP synthase complex which were obtained in BN gel band were all detected in 2-D gel. Furthermore, we were able to identify large, extremely hydrophobic proteins in BN gel by in-gel digestion and MS/MS analysis. In summary, this study is the first to apply the BN PAGE technique to the separation of potential BCG membrane protein complexes. Our results may provide some clues for BCG protein interaction studies and designing strategies against bacterial infection.

## Methods

### Bacterial cell cultivation

*M. bovis *var BCG NCTC 5692 from Beijing Tuberculosis and Thoracic Tumor Research Institute (Beijing, P.R. China) was cultivated in 5 liters of Sauton's liquid medium for 3 weeks at 37°C without shaking. Cells were harvested by centrifugation at 12,000 × *g *for 10 min at 4°C, and washed three times with ice-cold phosphate buffered saline (pH 7.4). Protease inhibitor cocktail (Roche, Germany) was added to all buffer solutions before use. The pelleted cells were frozen at -80°C prior to use.

### Sample preparation

All bacteria and sample manipulations were performed at 4°C. Membrane samples were prepared according to the method described by Sinha *et al. *with some modifications [7]. In brief, the cell pellet was probe-sonicated in ice-cold sonication buffer (50 mM Tris-HCl, l0 mM MgCl_2_, 0.02% sodium azide, pH7.4) containing protease inhibitor cocktail. The sonicates were centrifuged initially at 20,000 × *g *for 20 min to remove any unbroken cells and debris, and later at 150,000 × g for 90 min to obtain cell membrane sediment. The sediment was washed three times in sonication buffer containing protease inhibitor to remove cytosolic contaminants. The washed sediment was divided into four aliquots. The first two aliquots were suspended in sample buffer (750 mM ε-aminocaproic acid, 50 mM Bis-Tris, 0.5 mM EDTA-Na_2_, pH 7.0) and 10% DDM was added to a final concentrations of 2% and 1%, respectively. The samples were stirred slowly on ice for at least 1 h to solubilize the membrane fractions. The soluble proteins were collected by centrifugation at 12, 000 × *g *for 60 min at 4°C prior to electrophoresis. The other two aliquots were solubilized with 2% Triton X-100 to final concentrations of 0.2% and 0.1%, respectively. The subsequent samples were treated as described above prior to electrophoresis.

### BN PAGE and extraction of the peptide mixtures

Membrane samples were analyzed by BN PAGE according to the method of Schägger and von Jagow with minor modifications [17]. Briefly, a stacking gel of 3.5% and a separating gradient gel of 4-16% were used. Anode and cathode electrophoresis buffers were different as described previously [44]. About 80 μg protein from each aliquot was loaded to each lane and electrophoresis was conducted at 120 V for 2 h, and then adjusted to 200 V. After the blue running front has moved about half the desired total running distance, the blue cathode buffer was replaced with colorless BN cathode buffer for better detection of faint protein bands and the run was then continued until the front had reached the bottom of the separating gel. A high molecular weight calibration kit (GE Healthcare, UK) was used to indicate the protein size. Thyroglobulin (669 kDa), ferritin (440 kDa), catalase (232 kDa), lactate dehydrogenase (140 kDa) and bovine serum albumin (66 kDa) were used as markers.

After electrophoresis, gels were stained with colloidal Coomassie stain as described previously [45]. From the stained gels, nine bands with high staining intensity were chosen for excision and destaining. Proteins in each band were reduced with 10 mM dithiothreitol and alkylated with 55 mM iodoacetamide. In-gel trypsin digestion was then performed as described previously [46]. Tryptic peptides were then extracted by incubating each band with extraction solution (50 μl 1% TFA in 60% ACN) for 20 min at RT. The supernatant collected by centrifugation were concentrated and desalted with ZipTip C_18 _(Millipore, USA). The tryptic peptides were dried using a Speed-vac centrifuge (Eppendorf, Germany) and resolubilized in 0.1% TFA for subsequent 2-D LC-MS/MS analysis.

### LC separation and MS identification

Each distinct sample of 8 μl peptide mixture was loaded by the autosampler onto a reverse phase C_18 _PepMap 100, 3 μm, 100Å nano-column (75 μm i.d. × 15 cm) of Ultimate 3000 Nano and cap system (Dionex, USA). The flow rate was 2.0 μl/min for the nano-column, and the solvent gradient was 4% B to 60% B in 30 minutes, then 60-100% B in 5 minutes. Solvent A was aqueous 0.05% TFA and solvent B was aqueous 80% ACN in 0.04% TFA. Thirty seconds fractions of the LC flow were automatically spotted onto a Prespotted AnchorChip disposable targets using PROTEINEER fc (Bruker, Germany). Dried samples were washed with 10 mM NH4H2PO4 in 0.1% TFA (15 seconds) to provide matrix (α-cyano-4-hydroxycinnamic acid, HCCA) thin layer preparations for MALDI TOF/TOF analysis using an Ultraflex III spectrometer (Bruker, Germany).

MS measurements were performed in positive ion reflector mode with 20 kV accelerating voltage and 23 kV reflecting voltage, and spectra were calibrated using PeptideCalibStandard II (Bruker, Germany) of mass range 800-3200 Da as external standards [47]. WARP-LC 1.0 software controlled the precursor ion selection and triggered the MASCOT (Matrix Science) database search, and BioTools 3.0 was used for data visualization and post processing. All these software packages were provided by Bruker Daltonics GmbH, Germany.

### SDS PAGE and MALDI-TOF/TOF MS measurements

Nine bands corresponding to complexes with high staining intensity were excised from BN gel and incubated in 1% (w/v) SDS/1% (v/v) β-mercaptoethanol solution for 1 h. After a rapid rinse with water nine bands were loaded on top of a 5% stacking and 12% separating gel. The gap between the bands and the gel was filled with 1% low melt agarose. Electrophoresis was performed at 4°C, starting for 3 h at 80 V, and the voltage was then raised to 150 V overnight (max. 50 mA). Spots were excised from the Coomassie blue-stained gel and subjected to in-gel digestion protocol. The digestion mixture was dissolved in 2 μl 0.1% TFA prior to MALDI-TOF MS analysis. One microliter of the analyte solution, along with equivalent matrix solution (HCCA), was mixed and applied onto 600 μm AnchorChip target for MALDI-TOF/TOF analysis. MS measurements were performed as described above.

### Data Interpretation and Database Searching

The raw MS/MS data were searched against a composite target/decoy protein sequence database using the program MASCOT 2.1 http://www.matrixscience.com. The target component comprised 3,952 protein sequences derived from *M. bovis *BCG Pasteur 1173P2 (downloaded from the National Centre for Biotechnology Information database (NC_008769)) and protein sequences of known contaminant proteins, including trypsin and human keratin. Moreover, the decoy component was composed of the reversed sequences of all proteins in the target component. Search parameters allowed for up to two missed tryptic cleavage sites, the carbamidomethylation of cysteine, the possible oxidation of methionine, and monoisotopic mass type. Furthermore, peptide mass tolerance was ±0.2 Da and MS/MS tolerance was ±0.6 Da. For positive identification, the Mowse score of the result of (-10 × Log (P)) had to be over the significance threshold level (P < 0.05). Moreover, identification of individual proteins matched with two or more unique peptides was considered as valid identification.

### Bioinformatics analysis

The physicochemical characteristics of all identified proteins were analyzed by some software. The theoretical *M*r, p*I *value and average GRAVY score were obtained from the Swiss-Prot and TrEMBL databases http://us.expasy.org/tools/protparam.html. Predictions of transmembrane topology and possible signal sequence in the proteins were conducted using the TMHMM 2.0 program and SignalP program, respectively, both of which were publicly available from the Centre for Biological Sequence Analysis at the Technical University of Denmark http://www.cbs.dtu.dk/services. Functional association prediction between identified proteins was analyzed using STRING version 8.3 interaction database http://string.embl.de/. Functional classifications of the identified proteins were determined using the Pasteur Institute functional classification tree http://genolist.pasteur.fr/BCGList/.

## Abbreviations

ACN: acetonitrile; AG: arabinogalactan; BCG: bacillus Calmette-Guérin; BN: blue native; CBB: Coomassie Brilliant Blue; DDM: n-dodecyl-β-D-maltoside; 2-DE: two-dimensional gel electrophoresis; EMB: ethambutol; FadD: fatty acid degradation; GRAVY: grand average of hydrophobicity; Hsp: heat shock protein; IEF: isoelectric focusing; IMP: integral membrane protein; kbp: kilobase pair(s); kDa: kilodalton; LC-MS/MS: liquid chromatography tandem mass spectrometry; MALDI TOF: matrix-assisted laser desorption/ionization time of flight; *M*r: molecular mass; PAGE: polyacrylamide gel electrophoresis; PDIM: phthiocerol dimycocerosate; p*I*: isoelectric point; PKS: polyketide synthase; PPS: phenolpthiocerol synthase; RT: rome temperature; SDH: succinate dehydrogenase; SDS: sodium dodecyl sulphate; SL: sulfolipid; TB: tuberculosis; TFA: trifluoroacetic acid; TMH: transmembrane helix

## Authors' contributions

JZ performed collection of *M. bovis *BCG, samples preparation, the mass spectrometry experiments, data processing and bioinformatics analysis, and wrote the manuscript. CW and WL participated in the mass spectrometry experiments and data processing, and helped write the manuscript. LZ and LL contributed to samples preparation, the mass spectrometry experiments and bioinformatics analysis. WL carried out collection of *M. bovis *BCG and samples preparation. QJ designed the study, supervised the research and reviewed the manuscript. All authors read and approved the final manuscript.

## Supplementary Material

Additional file 1 **List of the identified proteins by MS analysis showing the sequence of all the identified peptides by independent MS/MS fragmentation**. The charge state of each peptide is +1. Accession: the identifier number from the National Centre for Biotechnology Information database; Protein ID: protein name in *M. bovis *BCG database; Score: the Mascot score of the protein identification; MW [kDa]: molecular weight in kDa of the respective protein; p*I*: isoelectric point of the protein, calculated from its amino acid sequence; SC[%]: sequence coverage in percent, calculated from the identified peptides of the respective protein; RMS [ppm]: the RMS value of the deltas between the calculated and experimental masses of the peptides, which belong to a particular protein; Inten^1^.: sum of all intensity values related to 100 laser shots for all peptides, which belong to a particular protein; S/N^1^: sum of all S/N-values for all peptides, which belong to a particular protein; MH+ (calc) [Da]: calculated mass of the singly protonated peptide; Δm [Da]: difference between the measured and calculated masses of the respective peptide in Dalton; Inten^2^: the sum of the MS peak intensities per 100 laser shots; S/N^2^: S/N ratio of the respective peptide; Sequence: the sequence of the corresponding peptide. Click here for file
